# Clinical responses to PD-1 inhibition and their molecular characterization in six patients with mismatch repair-deficient metastatic cancer of the digestive system

**DOI:** 10.1007/s00432-020-03335-2

**Published:** 2020-08-09

**Authors:** Daniela Hirsch, Timo Gaiser, Kirsten Merx, Simone Weingaertner, Michael Forster, Alexander Hendricks, Matthias Woenckhaus, Thomas Schubert, Ralf-Dieter Hofheinz, Deniz Gencer

**Affiliations:** 1 Institute of Pathology, Medical Faculty Mannheim, University Medical Center Mannheim, Heidelberg University, Theodor-Kutzer-Ufer 1-3, 68167 Mannheim, Germany; 2 Department of Medicine III, Medical Faculty Mannheim, University Medical Center Mannheim, Heidelberg University, Theodor-Kutzer-Ufer 1-3, 68167 Mannheim, Germany; 3 Institute of Clinical Molecular Biology, Christian-Albrechts-University Kiel, Kiel, Germany; 4 Department of Surgery, University Medical Center Rostock, Rostock, Germany; 5 Institute of Pathology, Caritas-Hospital Bad Mergentheim, Bad Mergentheim, Germany; 6 Institute of Applied Pathology, Speyer, Germany

**Keywords:** Immunotherapy, Microsatellite instability, Colorectal cancer, Tumor mutation burden, Pembrolizumab, Nivolumab

## Abstract

**Purpose:**

Immune checkpoint inhibitors have shown efficacy in patients with microsatellite instability-high/mismatch repair-deficient (MSI-H/dMMR) gastrointestinal (GI) cancers. However, depth and duration of clinical response is not uniform. We assessed tumor mutation burden (TMB) as a response marker in patients with GI cancers treated with immune checkpoint inhibitors.

**Methods:**

Detailed clinical and response data were collected from six patients with metastatic MSI-H/dMMR GI cancers treated with immune checkpoint inhibitors. Efficacy was assessed by Response Evaluation Criteria in Solid Tumors (RECIST) version 1.1. Tumors and matched normal tissue were profiled by targeted next generation sequencing (127 gene panel, size 0.8 Mb). Impact of included mutation types, germline filtering methodology and different variant allele frequency thresholds on TMB estimation was assessed.

**Results:**

Objective radiographic responses were observed in all six patients, and complete response was achieved in two of the six patients. Responses were durable (minimum 25 months). TMB estimates were clearly above the two recently reported cut-offs for metastatic colorectal cancer of 12 or 37 mutations per megabase for five of six patients, respectively, while one patient had borderline TMB elevation. TMB did not show an association with extent and duration of response but was influenced by included mutation types, germline filtering method and variant allele frequency threshold.

**Conclusion:**

Our case series confirms the clinical benefit of immune checkpoint blockade in patients with metastatic MSI-H/dMMR GI cancers and illustrates the vulnerability of TMB as predictive marker in a subset of patients.

**Electronic supplementary material:**

The online version of this article (10.1007/s00432-020-03335-2) contains supplementary material, which is available to authorized users.

## Introduction

In 2017, the U.S. Food and Drug Administration (FDA) granted approval to the programmed death receptor-1 (PD-1) blocking antibody pembrolizumab for patients with unresectable or metastatic, microsatellite instability-high (MSI-H) or mismatch repair-deficient (dMMR) solid tumors that have progressed following prior treatment and who have no alternative treatment options as well as for patients with MSI-H/dMMR metastatic colorectal cancer (mCRC) after failure of fluoropyrimidine, oxaliplatin, and irinotecan. This approval was recognized as a breakthrough given the fact that this was FDA’s first tumor site-agnostic approval.

Several uncontrolled studies including patients with MSI tumors originating from different sites (albeit mainly from the colon and rectum) reported a high rate of in part heavily pretreated patients deriving a clinical benefit of treatment with pembrolizumab or nivolumab ± ipilimumab in terms of long-lasting disease stabilization and tumor remissions (Le et al. 2017; Overman et al. 2017, 2018). More recently, results from 45 patients with MSI-H/dMMR mCRC treated 1st line with nivolumab plus low-dose ipilimumab were reported (Lenz et al. 2019). After a median follow-up of 13.8 months, response rate and disease control rate were 60% and 84%, respectively, and 12-month progression-free survival and overall survival rates were 77% and 83%, respectively.

Moreover, the use of checkpoint inhibitors administered only twice during the waiting period for curative surgery in early stage colon cancer led to complete pathological remissions in four of seven patients with MSI-H colon cancer, while none of eight patients with a microsatellite stable tumor exhibited signs of major pathohistological remission (Chalabi et al. 2018).

MSI-H tumors are characterized by an increased tumor mutation burden (TMB), exhibit increased expression of neoantigens and display higher numbers of tumor-infiltrating lymphocytes (Willis et al. 2020). Susceptibility to treatment with checkpoint inhibitors is consequently increased (Nebot-Bral et al. 2019). However, the presence of MSI-H/dMMR features does not guarantee benefit from checkpoint inhibitor treatment and deeper understanding of the landscape of tumor mutations and their potential predictive potential is mandatory, especially when checkpoint inhibitors are being used in earlier treatment lines with potential alternative treatment options. For instance, loss-of-function mutations in genes relevant for antigen presentation or immune response such as *JAK1*, *JAK2*, *B2M* and *STK11* have been identified as mediators of resistance to PD-1 inhibition despite overall high TMB (Shin et al. 2017; Skoulidis et al. 2018; Zaretsky et al. 2016).

Herein, we report on six patients with MSI-H/dMMR metastatic gastrointestinal (GI) cancers undergoing treatment with checkpoint inhibitors and along with their tumor mutational profile.

## Materials and methods

### Patients and eligibility criteria

We report on our first six consecutive patients for whom treatment with checkpoint inhibitors was initiated between June 2016 and August 2017. Patients suffered from progressive MSI-H/dMMR metastatic cancer of the digestive system (four patients with colon carcinoma, one patient with duodenal carcinoma, one patient with cholangiocarcinoma). All patients had received at least one prior therapy and had evidence of progressive disease prior to checkpoint inhibition. All patients were naïve to anti-PD-1, anti-PD-L1 and anti-PD-L2 antibodies. Molecular analysis by targeted next generation sequencing (127 gene panel, 0.8 Mb) was performed post-hoc. Treatment-naïve tumor tissue of the primary tumor (patients P1 to P4 and P6) or a metastatic lesion (P5) was used for molecular testing. The study procedure was approved by the Medical Ethics Commission II of Heidelberg University (Medical Faculty Mannheim; 2020-807R) including a waiver for informed consent.

### Treatment

Patients received either pembrolizumab (2 mg/kg every 3 weeks, maximum dose 200 mg) or nivolumab (3 mg/kg every 2 weeks). Treatment was generally continued until unacceptable toxicity, or disease progression. Serum biomarkers (CEA, CA19-9, CA72-4) were measured on an individual basis but generally at baseline and if elevated at baseline further monitored along with radiographic assessments. Radiographic assessments were performed every two to four months depending on patient performance and disease dynamics.

### Tumor sample collection for molecular analysis

Formalin-fixed paraffin-embedded (FFPE) tumor tissues were collected from the archives of the Institutes of Pathology in Mannheim, Bad Mergentheim and Speyer (all Germany). Histology was reviewed by two pathologists (DH, TG) and tumor areas containing at least 40% tumor cells were marked for molecular testing. Treatment-naïve tumor tissue (primary tumor for patients P1–P4 and P6, metastatic lesion for patient P5) was used for molecular testing.

### DNA isolation

DNA extraction of FFPE tumor and respective normal tissues was done as published previously (Hirsch et al. 2012). DNA concentration was measured by fluorometric quantitation (Qubit 3.0 Fluorometer, Life Technologies, Thermo Fisher Scientific, Carlsbad, CA, USA) using the Qubit dsDNA HS (High Sensitivity) Assay Kit (Life Technologies).

### Analysis of mismatch repair/microsatellite status

Mismatch repair/microsatellite status of tumors was determined by immunohistochemistry (IHC) and/or polymerase chain reaction (PCR) as described previously (Hirsch et al. 2018). Briefly, IHC was performed using the following primary antibodies: MLH1 (1:25; clone ES05, cat # M3640, Dako, Agilent Pathology Solutions, Agilent, Santa Clara, CA, USA), MSH2 (ready-to-use; clone FE11, cat # IR085, Dako), MSH6 (ready-to-use; clone EP49, cat # IR086, Dako), and PMS2 (1:50; clone EP51, cat # M3647, Dako). Detection was done using the EnVision Detection System, Peroxidase/DAB, Rabbit/Mouse (cat # K5007, Dako). IHC stainings were validated by internal and/or external positive controls as well as negative control specimens. IHC stainings were evaluated by two pathologists (DH, TG). Microsatellite PCR of tumor and corresponding normal DNA was done using a panel of five mononucleotide markers (BAT25, BAT26, NR-21, NR-24, and MONO-27; cf. MSI Analysis System, Promega), and a panel of two mononucleotide (BAT25 and BAT26) and three dinucleotide markers [D5S346, D2S123, and D17S250; so-called Bethesda panel; (Boland et al. 1998)]. Tumors were classified as MSI-H when two or more markers of either the Bethesda panel or the Promega panel showed an allelic size variation (i.e., a band shift compared with corresponding normal DNA).

### Targeted next generation sequencing

Targeted next generation sequencing (NGS) was done using the xGen Pan-Cancer Panel (v1.5; Integrated DNA Technologies, Coralville, IA, USA), spanning 0.8 Mb of the human genome and targeting 127 significantly mutated genes implicated across 12 tumor tissues (Kandoth et al. 2013). We determined the sequences of the tumors and matched normal (non-tumor) DNA for our six patients (matched normal DNA was not available for P5). Library preparation on FFPE isolated DNA was done using the Nextera DNA Exome library preparation kit (Illumina, San Diego, CA, USA) with 50 ng according to the manufacturer’s protocol, except for 14 instead of 10 PCR cycles. The resulting paired-end libraries were pooled using 100 ng of each library. The library pool was used for targeted capture with the xGen Pan-Cancer panel according to the manufacturer’s protocol except for 12 instead of 10 PCR cycles. An aliquot of 1.4 pM was sequenced on a NextSeq500 system (Illumina) using the 150 cycle mid-output kit (2 × 76 bps). Alignment was done using BWA mem 0.7.12-r1039 (Li and Durbin 2009) to hg19. The mean read depth for the targeted regions (mean coverage) was 1061X. We minimized calling FFPE artifacts by applying a minimal variant allele frequency (VAF) threshold of 10% (Melendez et al. 2018). Details on the bioinformatic analysis and variant calling are provided in the Supplementary Information.

### Calculation of tumor mutation burden (TMB)

TMB was calculated as the number of somatic coding mutations per megabase (Mb), including non-synonymous (missense) mutations, synonymous mutations, nonsense (stop) mutations, and/or frameshift mutations present above 10% VAF after filtering. Non-coding alterations and mutations predicted to be germline were not counted. We compared our data to the TMB thresholds suggested by Fabrizio et al. (Fabrizio et al. 2018) (≥ 11.7 mutations per Mb, synonymous and non-synonymous mutations) and Schrock et al. (Schrock et al. 2019) (proposed cut-off 37.4 mutations per Mb with a range of 37–41 mutations per Mb, synonymous and non-synonymous mutations), which both are based on the F1CDx Foundation medicine assay (324 genes, 1.11 Mb) and a VAF of 5% or greater (Frampton et al. 2013). Furthermore, influence of keeping or removing COSMIC listed variants on TMB estimation was evaluated. To assess the impact of VAF on TMB, we tested different cut-offs of 5%, 7.5%, 10%, 15%, and 20%, respectively. Impact of germline filtering was compared between (1) only computational germline filtering, (2) filtering against the matched normal sample, (3) filtering against the panel of other, non-matched normal samples (*n* = 4), and (4) filtering against the matched normal sample and a local panel of normal samples (Kiel normal samples; *n* = 55). Normal samples used as reference for germline filtering were processed in the same way as the tumor samples.

### Analysis of microsatellite status from targeted next generation sequencing data

To complement immunohistochemical and PCR-based MSI testing, we also assessed the microsatellite status from NGS data by applying MSIsensor on targeted NGS data from tumor and corresponding normal samples (Niu et al. 2014).

### Assessment of response, adverse events and survival times

Response and progression were evaluated using Response Evaluation Criteria in Solid Tumors (RECIST) 1.1 criteria (Eisenhauer et al. 2009; Schwartz et al. 2016). Toxicities were graded based on the National Cancer Institute Common Terminology Criteria for Adverse Events CTCAE, version 5.0, published by the National Cancer Institute/National Institutes of Health in November 2017). Progression-free survival (PFS) and overall survival (OS) rates were calculated using the Kaplan–Meier method. PFS was defined as the time from the date of the initial dose of immune checkpoint inhibition to the date of disease progression or the date of death due to any cause, whichever occurred first. PFS was censored on the date of the last evaluable tumor assessment documenting absence of progressive disease for patients who were alive and progression-free. OS was defined as the time from the initial dose due to death of any cause. For patients who were still alive at the time of analysis, the OS time was censored on the last date the patients were known to be alive. Duration of response was the time of first response as defined by RECIST 1.1 to the time of disease progression and was censored at the last evaluable tumor assessment for patients who had not progressed. Analyses and graphs were generated with GraphPad Prism software (version 8.4.2; San Diego, CA, USA).

## Results

### Patient, tumor and treatment characteristics

Our patient cohort consisted of six patients with mismatch-repair-deficient metastatic cancer of the digestive system (Table 1, Supplementary Table S1). Mismatch repair deficiency was assessed by polymerase chain reaction and/or immunohistochemistry. Cancers comprised three different entities: colorectal adenocarcinoma (4 patients), duodenal adenocarcinoma (1 patient) and cholangiocarcinoma (1 patient). All tumors were diagnosed between 2013 and 2016 in either locally advanced or metastatic stages (UICC stages IIIA-IV). Median age at diagnosis was 50 years (range 37–61). Four of the patients had a positive family history for gastrointestinal cancer and Lynch syndrome diagnosis was confirmed by genetic testing. Before immune checkpoint inhibitor therapy, all patients had received at least one prior line of chemotherapy and presented with metastatic progressive disease. PD-1 inhibition (nivolumab, pembrolizumab) for all patients was started between June 2016 and August 2017. Four patients were treated with pembrolizumab, while one patient received a combination of radiotherapy and nivolumab, and one patient nivolumab alone. Median time under PD-1 inhibition was 17 months (range 4–40). At the data cut-off, PD-1 inhibition is still ongoing in two patients, three patients had discontinued therapy due to excellent response with ongoing remission, and one patient had deceased from tumor progression. Median duration of response was 25 months (range  18–35 months). Patients were followed for a minimum of 25 months (median 27, maximum 40).

**Table 1:** Baseline patient and tumor characteristics

Patient ID	P1	P2	P3	P4	P5	P6
Age at diagnosis (years)	58	48	48	52	49	35
Gender	Male	Female	Male	Female	Male	Male
ECOG performance status	1	0	0	1	0	0
Primary tumor location	Colon (cecum)	Colon (transverse)	Colon (cecum)	Portal vein/bile duct/liver	Duodenum	Colon (ascending)
Tumor histology	Undifferentiated adenocarcinoma with signet ring cell component	Intestinal-type adenocarcinoma with mucinous component	Mucinous adenocarcinoma	Cholangio-carcinoma	Mucinous adenocarcinoma	Mucinous adenocarcinoma
Histologic differentiation	Poor	Moderate	Poor	Poor	Poor	Moderate
UICC stage at diagnosis	IV	IIIB	IIIB	IIIA	IV	IIIC
Metastatic sites	Peritoneum, abdominal lymph nodes	Liver, abdominal lymph nodes	Rectum	Bone, muscle	Duodenum, lung, abdominal lymph nodes	Liver, abdominal lymph nodes
Median time since initial diagnosis (months)	21	5	23	47	3	20
Previous primary tumor resection	Yes	Yes	Yes	Yes	No	Yes
Number of prior systemic treatments	2	1	1	1	1	1
Prior therapies received (ctx regimens/agents)	5-FU/FA + Bevacizumab (palliative 1st line) FOLFIRI + Bevacizumab (palliative 2nd line)	Capecitabine (adjuvant)	FOLFOX (adjuvant)	Gemcitabine + Cisplatin (palliative 1st line)	FOLFOXIRI (neoadjuvant)	FOLFOX + Bevacizumab (additive)
Prior radiotherapy	No	No	No	Yes	No	No
Previous malignancies	N/A	N/A	N/A	Endometrial carcinoma	N/A	N/A
Lynch syndrome	Yes	Yes	Yes	Yes	No	No
MMR status (IHC)	MMR-deficient	MMR-deficient	MMR-deficient	MMR-deficient	MMR-deficient	Not done
MMR protein expression (IHC)	MLH1 + , MSH2 −, MSH6 −, PMS2 +	MLH1 −, MSH2 + , MSH6 + , PMS2 −	MLH1 + , MSH2 −, MSH6 −, PMS2 +	MLH1 −, MSH2 + , MSH6 + , PMS2 −	MLH1 −, MSH2 + , MSH6 + , PMS2 −	N/A
MSI status (PCR)	MSI-high	Not done	Not done	MSI-high	Not done	MSI-high
*BRAF* mutation status	Wild-type	Wild-type	Not done	Not done	Not done	Wild-type
*KRAS* mutation status	p.A146T	p.G13D	Not done	Not done	Not done	Wild-type
*NRAS* mutation status	Wild-type	Wild-type	Not done	Not done	Not done	Wild-type
PD-L1 expression at baseline	Not done	< 1%	Not done	Not done	Not done	Not done

*IHC* immunohistochemistry; *MMR* mismatch repair; *MMR* mismatch repair; *MSI* microsatellite instability; *PCR* polymerase chain reaction

### Response evaluation, treatment duration and survival

**Fig. 1:**
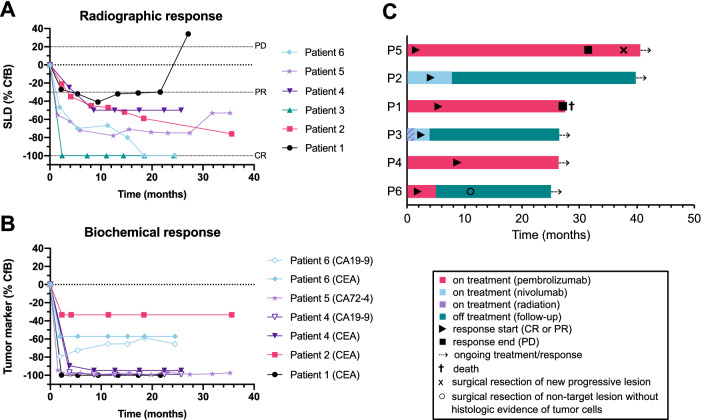
Clinical responses to PD-1 inhibition in six patients with mismatch repair-deficient metastatic carcinoma of the digestive system. **a** Spider plot of radiographic responses to PD-1 inhibition. Tumor responses were measured regularly; values show fractional change of the sum of lesion diameters from the baseline measurements of each measurable target lesion according to RECIST v1.1 criteria. **b** Spider plot of biochemical responses to anti-PD-1 treatment. Serum levels of protein biomarkers that were higher than the upper limit of normal at baseline were measured repeatedly, and the values represent relative changes from baseline. **c** Swimmer plot showing the time of objective response in relationship to duration of treatment and time of treatment cessation. *CfB* change from baseline; *CR* complete response; *PD* progressive disease; *PR* partial response; *SLD* sum of lesion diameter

Responses to PD-1 inhibition were evaluated radiographically in all six patients based on RECIST v1.1 criteria (Fig. 1a, Supplementary Figure S1A). In addition, biomarker levels for CA19-9, CA72-4 and/or CEA were monitored over the course of treatment if they had been elevated at baseline (Fig. 1b, Supplementary Figures S1B-D). Objective radiographic responses (i.e., partial or complete response based on RECIST v1.1 criteria) were noted in all six patients, with two patients achieving a complete response after 2.4 (P3) and 18.5 months (P6), respectively. The time to first objective radiographic response ranged from 1.5 to 8.6 months (median 2.3 months). In three patients (P1, P2, P4) tumor marker level reduction preceded objective radiographic response. However, the progression of patient P5 at 31.8 months after treatment initiation was first indicated by a radiographic increase in tumor size.

**Fig. 2:**
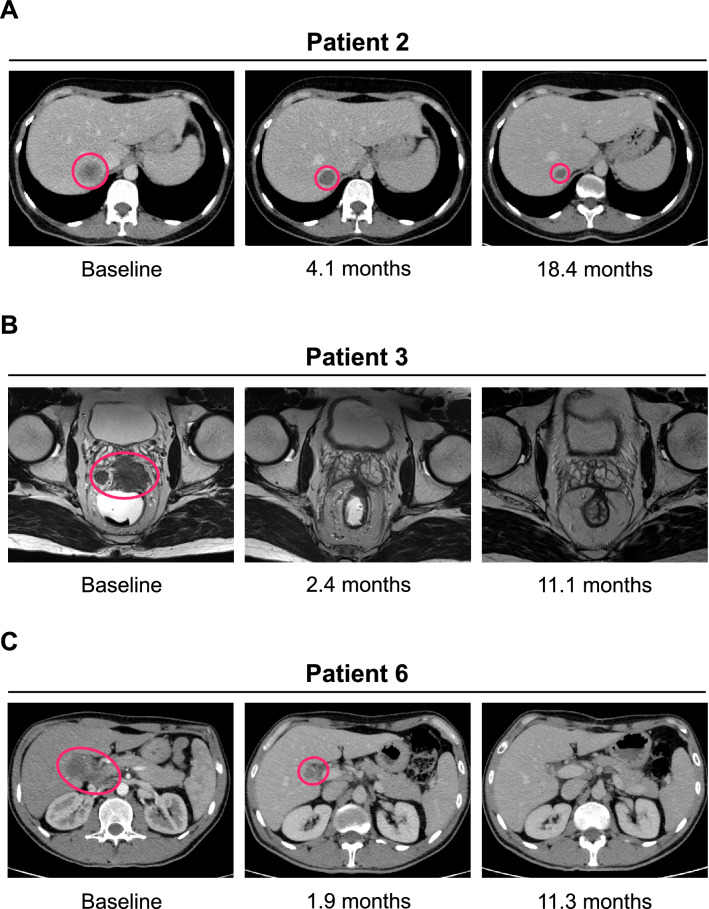
Radiographic response of the liver metastasis of patient 2 (**a**), pelvic metastasis of patient 3 (**b**), and liver metastasis of patient 6 (**c**). CT scans at baseline prior to PD-1 inhibition and follow-up CT scans are shown for each patient. Circled areas indicate the respective tumor lesions

Treatment was discontinued in three of six patients due to excellent response to anti-PD-1 therapy after 7.8 months (P2), 3.8 months (P3), and 4.9 months (P6), respectively, despite some residual disease by imaging in patients P2 and P6 at the time of treatment discontinuation (Fig. 1C and Fig. 2). As of the data cut-off, the time off therapy in these three patients was 31.1 (P2), 22.5 (P3) and 20.3 (P6) months, respectively. None of these three patients has shown evidence of cancer recurrence or progression since discontinuation of pembrolizumab or nivolumab so far. Instead, patient P6, who had partial response when taken off therapy, converted to complete response 13.6 months after treatment cessation.

Immunotherapy was continued in the other three patients whereby the dose for patient 1 was reduced to 4-weekly cycles after 13.3 months on treatment. Remarkably, the two disease progressions in our cohort (P1, P5) were observed in the patient group that continued with PD-1 inhibition. Patient 1 had the first evidence of progressive disease by imaging 27.1 months after starting PD-1 inhibition. Disease progressed rapidly and despite salvage therapy with FOLFOX, the patient passed away within one month after diagnosis of progressive disease. Patient 5 also had a progressing paraaortic lesion after 31.8 months of immunotherapy. The progressing metastatic lesion was excised by surgery and as of the data cut-off, which was 4.9 months after surgery, the patient is without tumor activity and PD-1 inhibition is continued. None of the patients had primary resistance to PD-1 inhibition, however, acquired resistance was noted in two patients, who developed progressive disease after an initial objective response to pembrolizumab. While one patient (P5) could be treated with local therapy (surgery) and survived and as of the data cut-off continues treatment with pembrolizumab, the other patient (P1) experienced rapid disease progression and died within one month after the first evidence of disease progression by imaging. Taken together, median progression-free survival (PFS) in our small cohort of six patients was 31.8 months (Supplementary Figure S2A); median overall survival (OS) has not yet been reached (median follow-up time of 29.9 months, range 25.2–40.2 months) (Supplementary Figure S2B). The estimates of PFS and OS at 1 and 2 years (12 and 24 months, respectively) were 100% (6 of 6 patients), respectively. The PFS and OS were not strikingly different in patients with colon cancer relative to those with the other GI cancer types. Neither PFS nor OS seemed to be influenced by tumors associated with Lynch syndrome. Adverse events during treatment were manageable and resembled those reported in other clinical studies using PD-1 inhibition (Le et al. 2015). No grade 3 or 4 events were noticed, in particular no immune-related adverse events leading to discontinuation of therapy were observed. Of note, patient P2 developed a reactive thymic hyperplasia under immunotherapy, which has still been present in the latest CT scan (Supplementary Figure S3). Detailed clinical history for each patient is provided in the supplement.

### Assessment of tumor mutation burden (TMB)

**Fig. 3:**
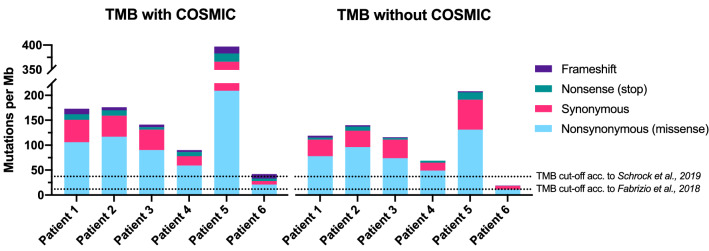
Tumor mutation burden (TMB) with and without COSMIC mutations. The chart illustrates the contribution of distinct mutation types to overall TMB levels. Shown are mutations with a variant allele frequency (VAF) of greater 10%. Filtering to remove germline variants and sequencing artifacts was against the matched normal sample and a local panel of normal samples (Kiel normal samples; *n* = 55). Indicated TMB thresholds from the studies of Schrock et al. and Fabrizio et al. are based on counts of nonsynonymous and synonymous mutations with a VAF threshold of 5%

In an attempt to better understand the genetic basis of the good responses to PD-1 inhibition in our small series of six patients, we performed targeted sequencing with a 127 gene panel spanning 0.8 Mb, which is considered suitable for TMB assessment according to current recommendations (Buttner et al. 2019). In addition to non-synonymous (missense), nonsense (stop) and frameshift mutations, we included synonymous mutations into our TMB calculation, following the rationale of Chalmers et al. (2017). Chalmers et al. (2017) reasoned that synonymous mutations, though they are not likely to be involved in creating immunogenicity, are a signal of mutational processes that will also have resulted in non-synonymous mutations and neoantigens elsewhere in the genome. Immunogenicity of particular mutation types and which mutation types should be included into TMB calculation is still a matter of debate and no uniform method exists. When using gene panels biased toward genes with functional mutations in cancer for TMB calculation, exclusion of mutations listed as known somatic alterations in COSMIC (Bamford et al. 2004) has been suggested (Chalmers et al. 2017). Overall, our data shows that MSI-H/dMMR leads to an increased TMB in all our patients, though to a different extent. When including COSMIC listed mutations, mutation counts averaged to 170 mutations per Mb with a range of 42–397 (Fig. 3). When excluding COSMIC listed mutations, an average of 112 mutations was detected per Mb with a range of 20–208. Hence, exclusion of COSMIC listed variants decreased absolute TMB levels by 19–53%, respectively. However, relative TMB levels of samples to one another remained similar.

The impact of included mutation types and COSMIC variants is critical, in particular with respect to patients with borderline TMB like patient P6 in our cohort, who despite borderline TMB had a durable response and clinical benefit from PD-1 inhibition via nivolumab, which is still ongoing after 25.2 months. In general, TMB levels did not associate with complete or partial response in our small subset of six patients. Moreover, patient P6 achieved complete response after 18.5 months, despite having the lowest TMB in our cohort. The other complete responder, patient P3, had an intermediate TMB level in our cohort. Apart from particular mutations that are included or not, TMB is influenced by a plethora of other factors (Stenzinger et al. 2019, 2020) including but not limited to minimum VAF threshold and method of germline filtering. As expected, the higher the VAF threshold, the lower the mutation count (Supplementary Figure S4). For VAFs below 10%, the method of germline filtering had a considerable impact on mutation counts. If only in silico germline filtering was performed, mutation counts were remarkably higher at VAFs of 5% and 7.5% compared to filtering against a matched normal sample or a local panel of normal samples (Supplementary Figure S4). This implies that thresholds below 10% may not be advisable for samples with solely computational germline filtering due to technical sequencing artifacts and FFPE artifacts, in line with Melendez et al. (2018). As filtering against a local panel of normal samples generated using the same workflow could at least partially resolve this issue, this should be considered as a practical option for routine molecular diagnostics where additional sequencing of a matched normal sample is often not feasible. Of note, for patient 1, who showed rapid progression, no potential genomic target causing this could be identified by the NGS analysis of the primary tumor sample. Due to rapid progression, tumor material from the progressing lesions could not be obtained.

### Assessment of microsatellite status by targeted next generation sequencing

To evaluate whether MSI status can reliably be assessed by panel sequencing, we applied the MSIsensor software tool (Niu et al. 2014) to our dataset (Supplementary Figure S5). This revealed an MSIsensor score highly suggestive for MSI-H in all five patients, for which a matched normal sample was available, a prerequisite for this tool. From a technical point of view, NGS data nicely confirmed MSI based on the MSIsensor score, making it a potentially useful tool for future studies and clinical purposes.

## Discussion

Checkpoint inhibitors have shown excellent efficacy in patients with MSI-H/dMMR GI cancers. For instance, in KEYNOTE-164, two cohorts of patients with pretreated mCRC received 3-weekly pembrolizumab (Le et al. 2020). Response rates were 33% in patients pretreated with ≥ 2 (cohort A) or ≥ 1 (cohort B) rounds of pretreatment, respectively. While median survival was 31.4 months in cohort A, it had not been reached in cohort B. Similarly, in CheckMate 142, patients with pretreated MSI-H/dMMR mCRC receiving monotherapy with nivolumab after ≥ 1 line of pretreatment exhibited a response rate of 31% and a 1-year survival rate of about 70% (Overman et al. 2017). However, despite these excellent response rates and long-term disease control, it has to be noticed that the rate of primary progression ranged between 26 and 46% in both studies. Preliminary data of 1st line patients receiving nivolumab in a cohort of the CheckMate 142 study reported 16% patients exhibiting primary progression (Lenz et al. 2019). This data underlines the need for additional biomarkers beyond MSI-H/dMMR to minimize patients exposed to checkpoint inhibitors instead of potentially active chemotherapy regimens, especially for those patients scheduled to receive checkpoint inhibitors in earlier lines of treatment.

Recently, Schrock and coworker (Schrock et al. 2019) suggested that TMB might serve as an additional biomarker in mCRC. In a cohort of 22 patients with mCRC treated with checkpoint inhibitors a cut-point of 37.4 mutations per Mb (range 37–41 mutations per Mb) for TMB was reported to distinguish between responders and non-responders. While all 13 patients with TMB values above this threshold exhibited long term benefit, 6 out of 9 patients with lower values showed primary progression. Their threshold is considerably higher than the 11.7 mutations per Mb threshold published by Fabrizio et al. (Fabrizio et al. 2018) in a previous study to identify MSI-high CRC samples. The stricter threshold suggested by Schrock et al. (Schrock et al. 2019) indeed leads to a more stringent identification of responders, however, at the expense of missing some potential responders since there is an overlap in mutation ranges between responders and non-responders. In other words, though recent evidence has shown that higher TMB scores are generally associated with improved response to immune checkpoint blockade across a wide variety of cancer types (Samstein et al. 2019), some patients still benefit from immune checkpoint blockade despite rather low mutation rates. This is evident in the cohort of Schrock et al. (3 patients) and in our cohort (1 patient; P6 with durable complete response to pembrolizumab). Another issue that needs to be considered when discussing TMB as a clinical biomarker in MSI-H patients, is the high variability and inconsistent reporting of (current) TMB assessment methods across different studies, which can create confusion for oncologists and may impact critical treatment decisions.

Originally, TMB was determined by whole exome sequencing (WES) and usually calculated as the number of non-synonymous mutations per exome or Mb reflecting the mutation load in all protein coding regions of the genome. Due to the increased interest in TMB for prediction of response to immune checkpoint inhibition and because WES is not yet routinely used in clinic, recent efforts have begun to validate TMB estimation based on targeted NGS panels, which are already implemented in routine molecular diagnostics for oncogenic mutation detection (Stenzinger et al. 2020). Currently, a minimum panel size of 0.8–1 Mb is widely accepted for TMB estimation for clinical purposes (Allgauer et al. 2018; Buttner et al. 2019), although in silico simulations based on TCGA exome data suggest a panel size of 1.5–3 Mb for an optimized cost–benefit ratio (Buchhalter et al. 2019). Overall, accuracy and precision of TMB estimation tend to increase with panel size (Garofalo et al. 2016) while below 0.5 Mb variance rises drastically, in particular for samples with low TMB (Chalmers et al. 2017). However, not only panel size matters but also panel composition as certain differences in TMB estimations have been observed between panels depending on their genomic composition (Xu et al. 2019). With the increased use of gene panels for TMB estimation, TMB definitions started to diverge from the original WES-based definition and other mutation types such as nonsense mutations, synonymous mutations and small indels were included, however, inconsistently across different laboratories and studies (Chan et al. 2019). Due to the enrichment of cancer relevant genes, it is suggested to remove oncogenic driver events by filtering against databases such as COSMIC (Bamford et al. 2004; Chalmers et al. 2017). As illustrated by our data, the mutation types included and removal of potentially oncogenic somatic mutations by exclusion of COSMIC-listed variants has a considerable effect on TMB estimation. However, COSMIC does not only contain oncogenic/cancer-relevant mutations but somatic mutations in general, a fact that may lead to an overcorrection of TMB estimated based on cancer gene panels. Furthermore, COSMIC is an evolving database, i.e., the number of cataloged mutations will increase over time, questioning its value for correcting panel-based TMB estimates for clinical purposes.

Two other important sources contributing to variability among TMB scores are the method of germline filtering and the minimum VAF threshold. Our data show that if no matched normal sample is sequenced along with the tumor sample, TMB estimation is more robust if filtered against at least a panel of normal samples (PON) that was processed in the same way as the case samples. The PON will not only help augment population frequency databases such as GnomAD, which are highly filtered and curated, but also help remove systematic and assay specific sequencing artifacts, which can be widespread even with matched normal samples. This is even more important when FFPE tissue is used as FFPE material is more artifact prone than fresh/frozen samples. Based on our observations, the commonly used VAF cutpoint of 5% (F1Dx, Oncomine Tumor Mutation Load Assay) may be too low and may thus increase the risk of including false positives, in particular when using FFPE tissue and only computational germline filtering. As filtering against a local PON could at least partially resolve this issue, this should be considered as practical option for routine molecular diagnostics.

Given the plethora of factors influencing exact TMB values (Stenzinger et al. 2019), exact TMB values are only comparable within individual studies using the exact same preanalytical workflow, sequencing methodology and bioinformatics pipeline. However, some confounding factors such as different levels of tumor cell purity, which significantly influence the number of called mutations even if the exact same workflow is followed, will remain in routine practice and are hard to control. For instance, harmonizing tumor purity would require disintegrating the tissue and enriching for the tumor cells by immunofluorescent markers. However, despite all the discrepancies and uncertainties regarding absolute TMB values, recent data from the QuIP study indicate a reasonable agreement of assignment to TMB categories between different laboratories and panels (Stenzinger et al. 2020). Apart from that, it is evident that the majority of responders with metastatic GI cancers greatly exceeds the currently suggested thresholds of 12 or 37 mutations per Mb, respectively, relatively independent from the specific gene panel used, mutation types included, and specific thresholds applied. The small group of patients with response to immune checkpoint inhibition and only low or borderline TMB, on the other hand, may need a biomarker other than TMB for identification. A potential option could be mutation signatures, which are a reflection of the underlying mutational processes, and other factors such as cell type composition/tumor-infiltrating lymphocytes (Loupakis et al. 2020). Ultimately, multi-omics testing may be the most reliable way to identify responders and non-responders to immune checkpoint inhibition. If TMB is pursued as a clinical biomarker for immunotherapy, consistent standards for TMB estimation and reporting are needed to minimize variability, to ensure reliable and reproducible identification of responders, and to allow comparison across studies (Fancello et al. 2019). To make informed clinical decisions, clinicians/oncologists need to be aware that different methods for TMB testing and reporting exist.

## Electronic supplementary material

Below is the link to the electronic supplementary material.

Supplementary file1 (PDF 1404 kb)
