# Supplementary material for: Clinical responses to PD-1 inhibition and their molecular characterization in six patients with mismatch repair-deficient metastatic cancer of the digestive system
Source: J Cancer Res Clin Oncol. 2020 Aug 9;147(1):263–73. doi: 10.1007/s00432-020-03335-2 (PMC7810640; doi:10.1007/s00432-020-03335-2)
Supplement: Supplementary file 1 — Supplementary file1 (PDF 1404 kb) [file 432_2020_3335_MOESM1_ESM.pdf]

Supplementary information

Detailed report of clinical response data for individual patients

Patient 1, a 58-year-old male with adenocarcinoma of the cecum, underwent right hemicolectomy shortly after being first diagnosed in April 2015. Surgery turned out to be highly complex and led to incomplete tumor resection (R2). Histopathologic assessment showed a signet ring cell carcinoma with involvement of lymphatic vessels and venules as well as eleven out of fourteen lymph nodes positive for tumor cells. The molecular genetic analysis showed *KRAS* mutation in exon 4. In June 2015, this patient started palliative first-line treatment with FOLFIRI plus anti-VEGF antibody bevacizumab and showed partial response in the performed imaging controls. In February 2016, therapy was switched to maintenance with 5-FU/FA and bevacizumab due to stable disease but then had to be escalated again to FOLFIRI plus bevacizumab in August 2016. Three months later, the CT scan showed massive progression of his abdominal lymph node metastasis with additional suspected peritoneal carcinomatosis, which led to initiation of checkpoint inhibition with anti-PD-L1 antibody pembrolizumab in common dose of 2 mg/kg body weight every three weeks. The imaging controls every three months showed initially partial remission of the documented target lesions under PD-L1 inhibition and since January 2017 stable disease. The level of tumor marker CEA (carcinoembryonic antigen) decreased since beginning pembrolizumab from 1198 µg/l to 3 µg/l in May 2017 (normal range 0 to 5 µg/l). No further increase of tumor marker was detected. In January 2018, the therapy interval was stretched to four weeks. Unfortunately, immunotherapy had to be discontinued in March 2019 due to progressive disease. The CT scan showed an abdominal lymph node bulk between the mesenteric vessels with suspected malignant ascites. The patient was admitted to hospital for pain management and evaluation of further treatment. A salvage therapy with FOLFOX did not lead to clinical or radiological benefit. The patient died in May 2019 in the hospital due to massive progression of his tumor disease.

Patient 2, a 48-year old female, was diagnosed with adenocarcinoma of the splenic flexure in March 2016. The performed clinical staging showed no further tumor involvement and the patient underwent left hemicolectomy shortly after. As a consequence of the post-operative pathological staging with tumor growth through the visceral peritoneum (pT4a) but without lymph node metastases and without lymphatic or blood vessel infiltration, the patient received adjuvant treatment with capecitabine as monotherapy. Only three months after beginning of adjuvant treatment, the imaging control showed suspected abdominal lymph nodes next to the mesenterium and the left kidney, and one liver metastasis up to 3 cm of diameter. The initiated treatment with PD-1 inhibitor nivolumab led to rapid shrinkage of the target lesions in the imaging controls. The liver metastasis showed cystic and necrotic transformation and no further tumor activity was noted since the second CT scan in November 2016; the abdominal lymph node metastases decreased to a size <1 cm. As a possible side effect of the immunotherapy, a mediastinal mass was discovered in November 2016, which was diagnosed as reactive thymic hyperplasia after checkpoint inhibitor therapy. No clinical symptoms associated to the thymic enlargement were reported by the patient. Also, no other side effects or therapy-specific toxicities were observed. Due to the excellent response, the checkpoint inhibition therapy was discontinued in March 2017 with ongoing remission in the latest imaging control in June 2019.

Patient 3, a 48-year-old male, was diagnosed with carcinoma of the cecum in September 2015 and received right hemicolectomy. Due to locally advanced stage IIIB (pT4a, N1 (1/25), L1, G3), adjuvant chemotherapy with FOLFOX was started in October 2015 and completed without major toxicities six months later. The first year of follow-up showed no tumor-associated events, but in July 2017 a new rectal tumor with measured size of 3.9 × 3.5 cm as well as locoregional suspected lymph nodes were found in the MRI scan. In the tumor conference, a rectal metastasis of the initial tumor disease or a metachronous secondary malignancy were discussed as differential diagnosis. In this case, an innovative therapeutic approach with neoadjuvant simultaneous radio-immunotherapy with radiation of the tumor area and the pelvic

lymph vessels plus nivolumab every two weeks was chosen as a potentially curative treatment. The patient underwent the treatment without major side effects with application of complete radiation dose of 50.4 Gy and also without any dose adjustments of nivolumab. After four weeks of simultaneous radio-immunotherapy, nivolumab was administered for another eight weeks alone until November 2017. The first follow-up with MRI and rectal endosonography after end of radio-immunotherapy showed a complete remission of the tumor manifestation. Therefore, planned tumor resection was canceled and therapy with nivolumab was discontinued within a watch and wait concept and continuous follow-ups. The last follow-up including endoscopy and MRI scan in July 2019 showed no further tumor manifestation.

Patient 4, a 52-year old female, was diagnosed with a cholangiocarcinoma in 2013. The patient then underwent extended hemihepatectomy of the right liver lobe in a complex eight-hour surgery due to infiltration of the portal vein. The histopathological staging showed locally advanced stage but no lymph node metastases. In accordance with the decision of the tumor conference, no adjuvant treatment was given. After an interval of two years with non-suspicious follow-up results, a 1.1 × 1.6 cm lump in the upper left pulmonary lobe was found in the imaging examination. Without any other signs of tumor activity, the decision for video-assisted thoracoscopy lobectomy was made. The procedure was done without any complications. The histological result confirmed a metastasis of the former cholangiocarcinoma. None of the resected lymph nodes showed tumor infiltration. One month later the patient reported an increasing pain of the right upper leg. The further diagnostic workup showed an isolated bone metastasis in the right greater trochanter. Primary resection of the metastasis with additional proximal femoral nailing was done in March 2016, followed by post-operative radiation of the right femur up to 30 Gy. During the following nine months, an increasing activity of the tumor disease could be observed. After radiotherapy of another bone metastasis in the left hip, a solitary cerebellar metastasis was diagnosed in November 2016 which was removed by neurosurgery followed by high-dose radiation (Gamma-Knife) of the tumor bed plus whole brain irradiation. In March

2017, the symptom burden increased due to progression of the bone metastases and a new intra-muscular metastasis in the right upper leg. Palliative first-line treatment with cisplatin and gemcitabine was started to control the progressive tumor disease. However, only four months after the beginning of palliative chemotherapy, another progression of the metastatic disease was documented, which led to change of therapy to pembrolizumab in August 2017. The first imaging control in November 2017 showed stability of the bone lesions and remission of the intra-muscular metastasis of the right leg and the surrounding tissue reaction, which remained stable until the last imaging follow-up in September 2019. The tumor markers CEA and CA19-9 rapidly decreased under checkpoint inhibition from initially 9032 kU/l (CA19-9; normal range 0 to 37 kU/l) and 19 µg/l (CEA; normal range 0 to 5 µg/l) to a minimum of 50 kU/l and 1 µg/l in July 2018. Since then, a slight increase of CA19-9 level to recently 91 kU/l has been observed without any clinical or radiological signs of tumor progression. Hence, immunotherapy is continued.

Patient 5, a 49-year-old male, was admitted to hospital due to weight loss in March 2016. The diagnostics showed locally advanced high-grade adenocarcinoma of the small bowel with long-segment infiltration of the duodenum and at least two lymph node bulks, one next to the pancreas up to 7 cm, another at the hepatic portal system up to 3 cm in diameter. Due to the non-resectable situation, the decision for neoadjuvant chemotherapy with potentially secondary curative treatment was made by tumor conference. From March to April 2016, the patient received neoadjuvant treatment with cumulative three cycles of FOLFOXIRI regimen. Unfortunately, the patient developed a clinically significant ileus and intestinal bleeding, which led to urgent salvage operation with palliative gastrojejunostomy. Treatment with pembrolizumab in common dose of 2 mg/kg bodyweight was started three weeks after surgery. Target lesions were the primary tumor, local peritoneal carcinomatosis, suspected metastasis of the lung as well as the abdominal lymph node bulks. A good partial response had already been observed in the first imaging control six weeks after initiation of treatment with pembrolizumab. An endoscopic control of the initial tumor localization in the region around the anastomosis in October 2017 showed no

macroscopic signs of residual tumor, which was confirmed by the histological analysis of the gathered tissue samples. However, the analysis of an ulcerating area close to the anastomosis revealed a florid anastomositis. It will remain elusive if there was causality between the inflammation and the immunotherapy. Until January 2018, the patient was in partial remission. The following imaging controls then showed stability of the target lesions. The CT scan in January 2019 showed discrete progression of a paraaortic lymph node from 17 mm to 25 mm with further signs of tumor progression in the following imaging in May 2019 showing dilatation of the duodenum due to the lymph node metastasis. Decision of the tumor conference was resection of the lymph nodes and part of the duodenum. Immunotherapy was continued after the successful operative intervention due to excellent tolerability of treatment with the aim of tumor control. At time of this analysis, there was no further tumor activity while immunotherapy was ongoing.

Patient 6, a 35-year-old male, was diagnosed with carcinoma of the ascending colon in January 2016. The pathology report after right hemicolectomy showed advanced stage with infiltrative growth of the tumor up to the abdominal wall, 15 affected lymph nodes (out of 51) and suggested microscopically incomplete resection (R1) because of detection of isolated tumor cells at the resection margin. The patient then received additional chemotherapy with FOLFOX and bevacizumab from March until August 2016. Initially, the follow-up was without events. In February 2017, the sonography showed a single liver metastasis with a diameter of 9 mm. For the next six months, the patient got lost to follow-up and presented himself in August 2017 with progression of the central liver metastasis, suspected retroperitoneal lymph nodes and probable tumor growth around the celiac artery/trunk. Immunotherapy with pembrolizumab was started in August 2017 and well tolerated without any treatment related event. The first two radiological controls in October 2017 and February 2018 showed partial remission after cumulative eight cycles of pembrolizumab. After tumor conference, the patient was admitted to surgery for a potentially secondary curative approach. In March 2018, an explorative laparotomy was conducted. The intraoperative sonography did not confirm any suspect of intrahepatic metastases,

thus a local lymphadenectomy of suspected interaortocaval lymph nodes and in the area of the hepatic portal system was performed, including cholecystectomy due to macroscopic tumor suspicion. The histopathological analysis showed complete pathological response without evidence of any vital tumor cells. The patient recovered from surgery without complications and has been followed up every three months, yet without any sign of tumor recurrence.

Details on bioinformatic analysis and variant calling

Initial variant calling was done using GensearchNGS (version 1.6.84, Phenosystems, Wallonia, Belgium) with the following filter criteria: minimum (tumor allele) frequency 3%, minimum coverage 140, minimum variant reads 2, ignore 5 bases from read borders, minimum alignment quality 20, minimum base quality 20, masked with ROI file xgen-pan-cancer-targets.bed. Next, technical filtering was done using the strand bias filters, VarBalance and PosBalance, i.e., forward/reverse read balance (1 is best, 0 is worst) >0, the alternative allele frequency filter >0.10 to minimize FFPE artifacts, and the gene name filter to exclude *CRIPAK* which attracts multiple mapping reads. In analogy to the settings used for PCR-based amplicon sequencing (Xu 2018), no deduplication filter was used. Deduplication favors wild-type sequence deduplication and thus would lead to disproportional amplification of minor allele frequency (mutations, artifacts) at deep coverages for our transposase-based Illumina Nextera library preparation method, which is based on enzymatic cutting of DNA at recurring motifs. After sequence alignment, there are stacks of sequences with identical start and end coordinates, strongly resembling PCR-amplicon libraries. For tumor samples with patient-matched normal samples, mutations not present in the normal but present in the tumor sample were obtained using GenSearch NGS Sample Filtering with options: consider filtered, require valid. For tumor samples without patient-matched normal samples, mutations were excluded that were detected at least once in the normal sample from the other five patients. An optional check for recurrent bioinformatic artifacts or non-pathogenic variants was performed against a local database of 55 fresh normal samples from fresh frozen DNA. The local database

was curated by excluding common polymorphisms that had a population frequency >1% in the Ensembl database as used by GenSearchNGS. Recurrent bioinformatic artifacts or non-pathogenic variants were taken to be those variants with a frequency of at least 4 of 55 in our local database. The resulting technically filtered variants were exported in vcf format and imported into the Agilent Alissa Interpret platform for annotation (COSMIC, gnomAD) and subsequent TMB calculation.

Reference

Xu C (2018) A review of somatic single nucleotide variant calling algorithms for next-generation sequencing data Comput Struct Biotechnol J 16:15-24 doi:10.1016/j.csbj.2018.01.003

Supplementary Table

Supplementary Table S1. PD-1 inhibition and outcomes in six patients with MMRd/MSI-H metastasized cancer of the digestive system.

Patient ID	P1	P2	P3	P4	P5	P6
**Primary tumor site**	Colon	Colon	Colon	Portal vein/bile duct/liver	Duodenum	Colon
**Metastatic sites**	Peritoneum, abdominal lymph nodes	Liver, abdominal lymph nodes	Rectum	Bone, muscle	Duodenum, lung, abdominal lymph nodes	Liver, abdominal lymph nodes
**Anti-PD-1 therapy**	Pembro-lizumab	Nivolumab	Nivolumab/ radiation	Nivolumab	Pembro-lizumab	Pembro-lizumab
**Line of therapy**	3	2	2	2	2	2
**MMR status by IHC**	dMMR	dMMR	dMMR	dMMR	dMMR	Not done
**MSI status by PCR**	MSI-H	Not done	Not done	MSI-H	Not done	MSI-H
**TMB (mut/Mb)#**	173	176	143	89	397	42
**MSIsensor Score (%)**	51.8	55.8	52.7	33.9	N/A	53.6
**Best overall response**	PR	PR	CR	PR	PR	PR
**PFS (months)**	27.1	38.9*	26.3*	26.2*	31.8	25.2*
**OS (months)**	28.9	38.9*	26.3*	26.2*	40.2*	25.2*

*ongoing; TMB defined as non-synonymous (missense), synonymous, frameshift, and nonsense (stop) mutations with VAF > 0.10 including COSMIC listed mutations. TMB, tumor mutation burden; MMR, mismatch repair; dMMR, mismatch repair deficient; MSI-H, microsatellite instability high; MSI, microsatellite instability; CR, complete response; PR, partial response; PFS, progression-free survival; OS, overall survival.

Supplementary Figures

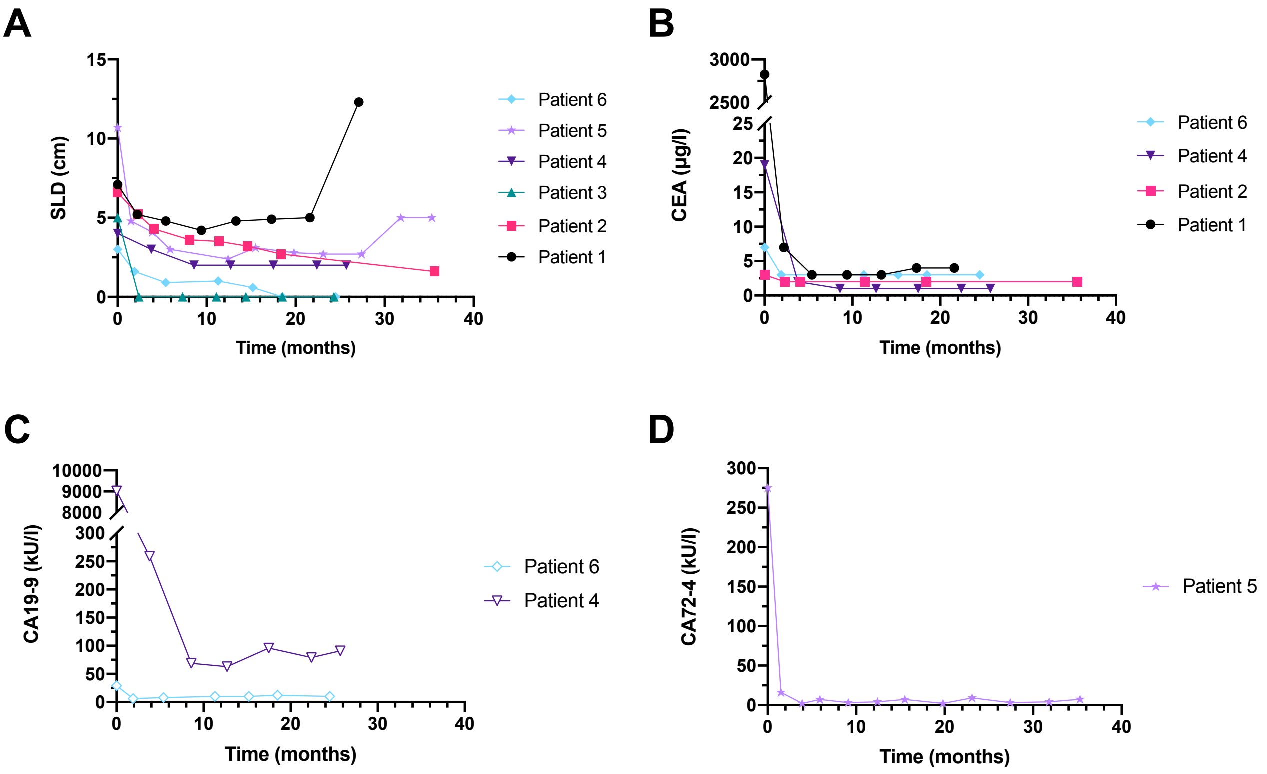

Supplementary Figure S1. Spaghetti plots representing the time course of individual raw SLD values (A) and serum biomarker levels (B to D). SLD, sum of lesion diameter.

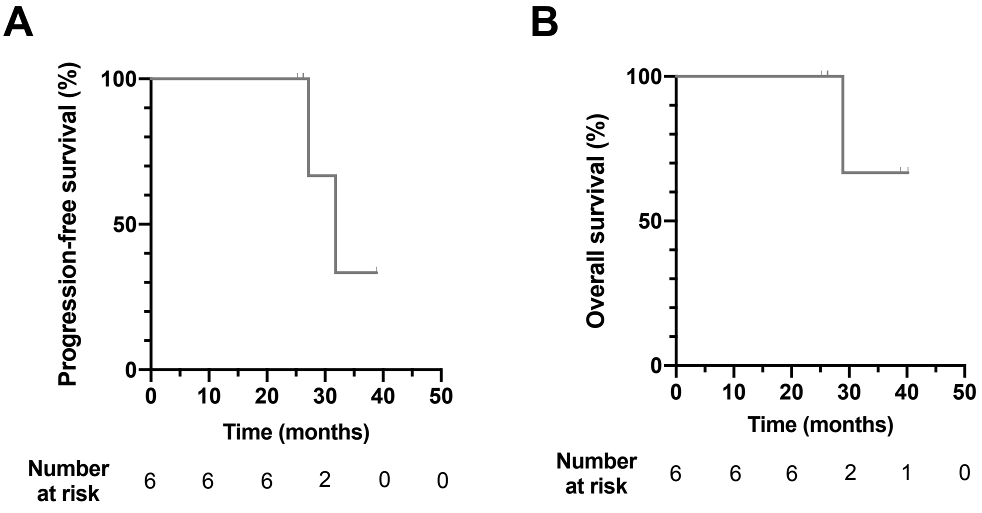

Supplementary Figure S2. Clinical benefit of PD-1 inhibition in metastatic MSI-H/dMMR gastrointestinal cancers. Kaplan–Meier curves are shown for progression-free survival (A) and overall survival (B). Patients had a median progression-free survival of 31.8 months. Median overall survival has not yet been reached.

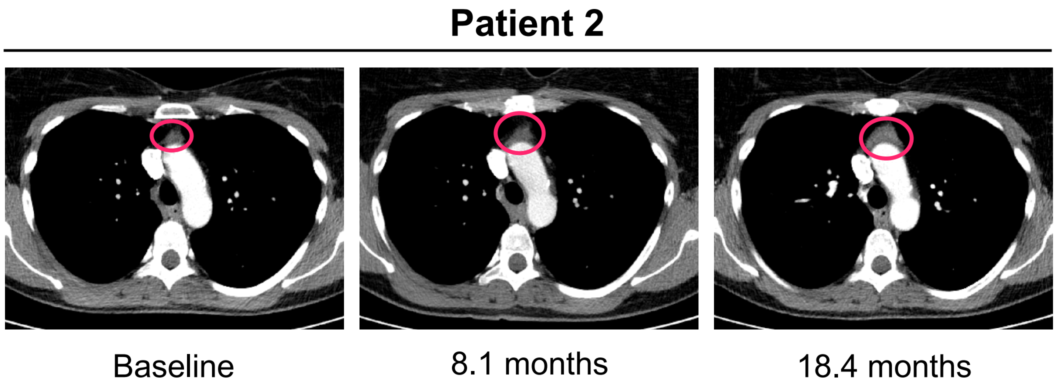

Supplementary Figure S3. Radiographic images of thymic hyperplasia under PD-1 inhibiton in patient 2. CT scans at baseline prior to PD-1 inhibition and follow-up CT scans are shown. Circled areas indicate the enlarged thymus.

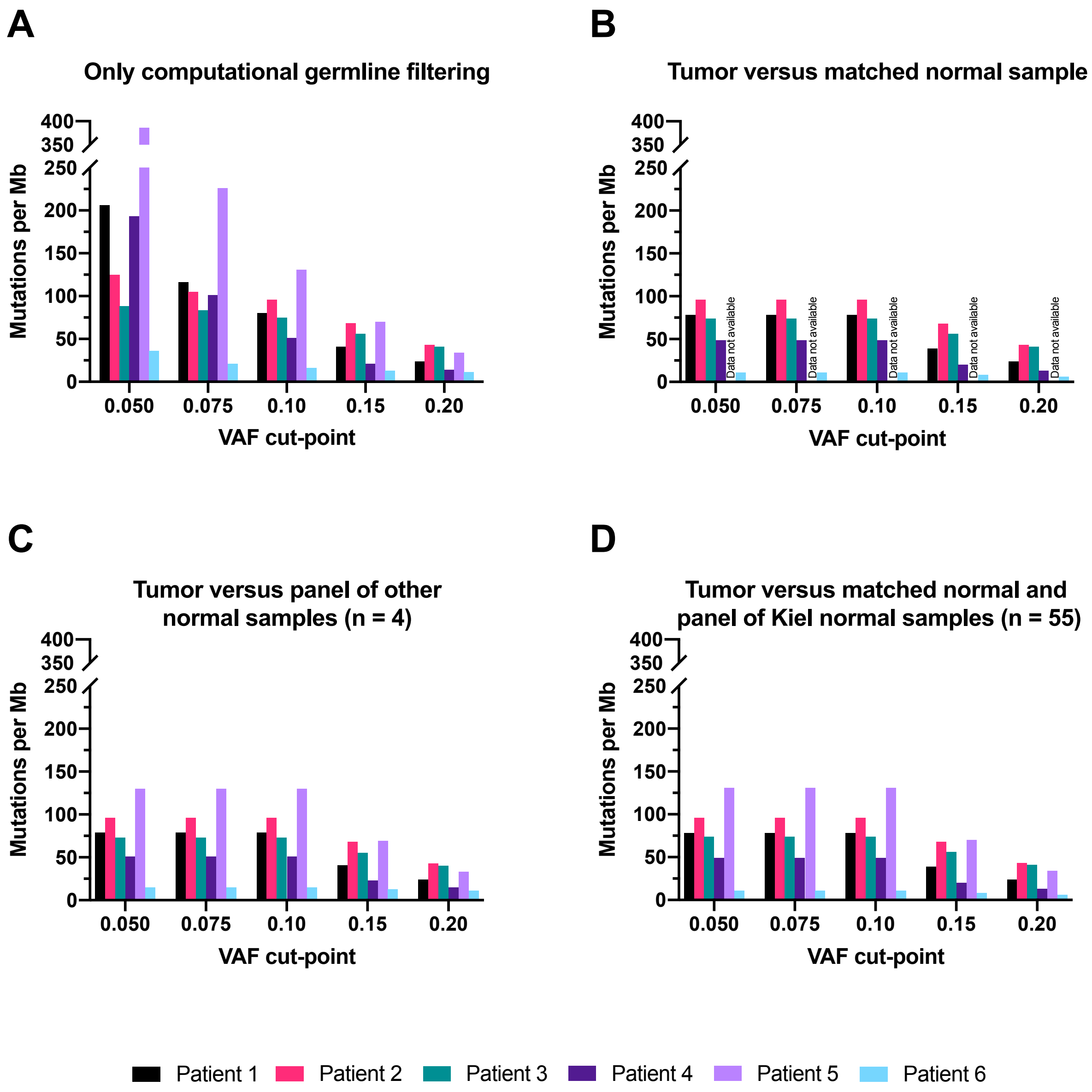

Supplementary Figure S4. Influence of different methods for germline filtering and different VAF cut-points on TMB. (A) Only computational germline filtering, (B) filtering against the matched normal sample, (C) filtering against the panel of other, non-matched normal samples (n = 4), and (D) filtering against the matched normal sample and a local panel of normal samples (Kiel normal samples; n = 55). Normal samples used as reference for germline filtering were processed in the same way as the tumor samples. All calculations are based on nonsynonymous, non-COSMIC mutations. Filtering against matched normal and/or a similarly processed local panel of normal samples reduces false positive somatic calls including technical artifacts and FFPE artifacts, especially at VAFs below 0.10. TMB, tumor mutation burden; VAF, variant allele frequency.

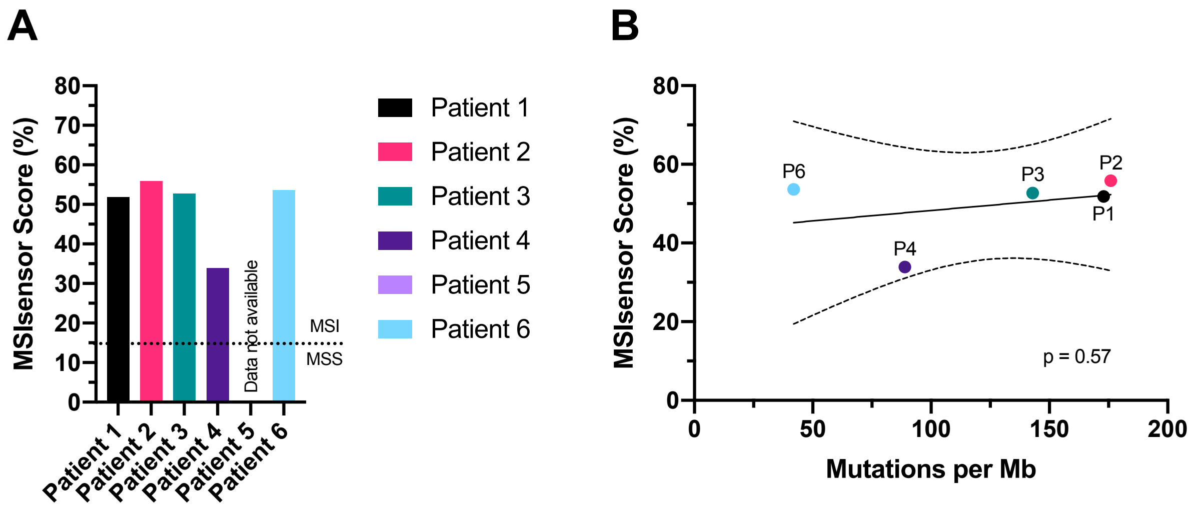

Supplementary Figure S5. MSIsensor-based detection of microsatellite instability using paired tumor-normal sequence data. (A) MSIsensor Score (y axis) represents the percentage of microsatellites with a somatic indel in sequencing data of tumor and respective normal tissue for each of the patients. For patient 5, MSIsensor analysis was not possible due to lack of normal tissue. MSI-H tumors are expected to have an MSIsensor Score above 15%. Reassuringly, all our samples have an MSIsensor Score consistent with MSI-H. (B) Scatter plot of MSIsensor Score versus tumor mutation count, showing no correlation ($r = 0.35$, $p_{slope=0} = 0.57$). An overall linear regression line is plotted along with the 95% confidence interval.
